# Phosphodiesterase 9A Inhibition Facilitates Corticostriatal Transmission in Wild-Type and Transgenic Rats That Model Huntington’s Disease

**DOI:** 10.3389/fnins.2020.00466

**Published:** 2020-06-03

**Authors:** Shreaya Chakroborty, Fredric P. Manfredsson, Alexander M. Dec, Peter W. Campbell, Grace E. Stutzmann, Vahri Beaumont, Anthony R. West

**Affiliations:** ^1^Department of Neuroscience, Rosalind Franklin University of Medicine and Science, North Chicago, IL, United States; ^2^Parkinson’s Disease Research Unit, Department of Neurobiology, Barrow Neurological Institute, Phoenix, AZ, United States; ^3^CHDI Management/CHDI Foundation, Los Angeles, CA, United States

**Keywords:** phosphodiesterase 9A, Huntington’s disease, corticostriatal transmission, medium spiny neurons, fast-spiking interneurons

## Abstract

Huntington’s disease (HD) results from abnormal expansion in CAG trinucleotide repeats within the HD gene, a mutation which leads to degeneration of striatal medium-sized spiny neurons (MSNs), deficits in corticostriatal transmission, and loss of motor control. Recent studies also indicate that metabolism of cyclic nucleotides by phosphodiesterases (PDEs) is dysregulated in striatal networks in a manner linked to deficits in corticostriatal transmission. The current study assessed cortically-evoked firing in electrophysiologically-identified MSNs and fast-spiking interneurons (FSIs) in aged (9–11 months old) wild-type (WT) and BACHD transgenic rats (TG5) treated with vehicle or the selective PDE9A inhibitor PF-04447943. WT and TG5 rats were anesthetized with urethane and single-unit activity was isolated during low frequency electrical stimulation of the ipsilateral motor cortex. Compared to WT controls, MSNs recorded in TG5 animals exhibited decreased spike probability during cortical stimulation delivered at low to moderate stimulation intensities. Moreover, large increases in onset latency of cortically-evoked spikes and decreases in spike probability were observed in FSIs recorded in TG5 animals. Acute systemic administration of the PDE9A inhibitor PF-04447943 significantly decreased the onset latency of cortically-evoked spikes in MSNs recorded in WT and TG5 rats. PDE9A inhibition also increased the proportion of MSNs responding to cortical stimulation and reversed deficits in spike probability observed in TG5 rats. As PDE9A is a cGMP specific enzyme, drugs such as PF-04447943 which act to facilitate striatal cGMP signaling and glutamatergic corticostriatal transmission could be useful therapeutic agents for restoring striatal function and alleviating motor and cognitive symptoms associated with HD.

## Introduction

Huntington’s disease (HD) is a dominantly inherited progressive neurodegenerative disorder caused by a polyglutamine (CAG) expansion within the gene encoding for the huntingtin protein (HDCRG, 1993). Hallmark pathological features of HD include progressive motor dysfunction characterized by an initial hyperkinetic phase (uncontrolled dance-like movements termed chorea), and a subsequent hypokinetic phase associated with dystonia and rigidity (Berardelli et al., 1999; Zuccato et al., 2010). Decline in motor control is often accompanied by cognitive, emotional, and psychiatric disturbances (Walker, 2007; Bates et al., 2015; Espinoza et al., 2019; Ellis et al., 2020). Neuropathological studies point to preferential degeneration of striatopallidal medium-sized spiny neurons (MSNs) and dysfunction of corticostriatal projection neurons (Wong et al., 1982; Reiner et al., 1988; Vonsattel et al., 2008). Striatal GABAergic interneurons are also compromised in HD models, particularly the fast-spiking parvalbumin expressing interneurons (FSIs) (Reiner et al., 2013), as well as the low threshold-spiking interneurons which colocalize neuronal nitric oxide synthase (nNOS), neuropeptide Y, somatostatin, and GABA (Holley et al., 2019). Electrophysiological studies of transgenic rodent models of HD have demonstrated a profound impairment in corticostriatal transmission characterized by imbalances in glutamate and GABA transmission, disruptions in striatal network activity, and abnormal uncoordinated MSN firing activity (Cepeda et al., 2007; Miller et al., 2010; Andre et al., 2011; Heikkinen et al., 2012; Indersmitten et al., 2015; Bunner and Rebec, 2016; Raymond, 2017).

Interestingly, perturbation of striatal cyclic nucleotide signaling and metabolism (Cramer et al., 1981; Luthi-Carter et al., 2000; Gines et al., 2003; Hebb et al., 2004; Hu et al., 2004; Leuti et al., 2013; Beaumont et al., 2016) and reduced hippocampal cyclic guanosine monophosphate (cGMP) levels (Saavedra et al., 2013; Puigdellivol et al., 2016) have been frequently observed in rodent models of HD. The synthesis of cGMP is largely dependent on nitric oxide (NO)-mediated activation of soluble guanylyl cyclase (sGC). The NO-sGC-cGMP pathway regulates multiple intracellular targets including protein kinase G (PKG), cyclic nucleotide gated channels (CNGCs), and cyclic nucleotide phosphodiesterases (PDEs) (Padovan-Neto and West, 2017). These signaling molecules play a central role in coordinating intracellular responses critically involved in neuromodulation of corticostriatal and hippocampal synaptic transmission and neuroplasticity (reviewed in Di Filippo et al., 2007; Puigdellivol et al., 2016). Electrophysiological and biochemical studies show that excitatory glutamatergic synaptic transmission in MSNs is initially facilitated by the activation of cGMP signaling (West and Grace, 2004; Nishi et al., 2008; Threlfell et al., 2009; Sammut et al., 2010; Padovan-Neto et al., 2015; Beaumont et al., 2016). Thus, pharmacological or genetic manipulations which attenuate NO-cGMP signaling cascades in MSNs exert an inhibitory influence on corticostriatal transmission (Threlfell and West, 2013; Hoque et al., 2017).

Given the primary role of PDEs in the metabolism of cAMP and cGMP, isoform selective PDE inhibitors have been advanced as therapeutic agents for slowing disease progression in preclinical models of HD (Giampa et al., 2009a, b, 2010) and alleviating motor and cognitive symptoms in HD (Beaumont et al., 2016). In support of this, inhibition of the cAMP-specific enzyme PDE4 blocks the sequestration of CREB binding protein, and rescues striatal FSIs and motor deficits in R6/2 mice (Giampa et al., 2009a). Additionally, PDE10A inhibitor treatment attenuates striatal and cortical pathology in R6/2 mice (Giampa et al., 2010). Elevations in cyclic nucleotide levels and CREB phosphorylation induced following PDE10A inhibition were also found to augment corticostriatal transmission, rescue hippocampal LTP, and reverse basal ganglia dysfunction in the R6/2 and Q175 HD mouse models via cAMP and cGMP-dependent mechanisms (Beaumont et al., 2016). Furthermore, the PDE5 inhibitor sildenafil increased hippocampal cGMP levels and improved memory in R6/1 mice (Puigdellivol et al., 2016).

Taken together, the above studies indicate that pharmacological targeting of specific isoforms of PDEs may effectively restore disruptions in cyclic nucleotide signaling and downstream targets, and potentially, dysfunctional neurotransmission in corticostriatal and hippocampal pathways in HD (Wild and Tabrizi, 2014; Mrzljak and Munoz-Sanjuan, 2015; Puigdellivol et al., 2016). The cGMP-specific enzyme PDE9A exhibits moderate levels of expression in human and rodent cortical, striatal, and hippocampal projection neurons, yet possesses by far the highest affinity (*Km* = 170 nM) for cGMP among all PDE isoforms (Fisher et al., 1998; Francis et al., 2006; Verhoest et al., 2009; Kleiman et al., 2012). Inhibitors of PDE9A robustly elevate striatal, cortical, and CSF cGMP levels and improve cognitive and sensory-motor gating performance on hippocampal and striatal-mediated tasks (Verhoest et al., 2009; Kleiman et al., 2012; Nagy et al., 2015). Moreover, the selective PDE9A inhibitor PF-04447943 has recently been shown to improve auditory gating in the cortex and hippocampus of BACHD transgenic (TG5) rats (Nagy et al., 2015). To determine the potential utility of PDE9A inhibition for restoring corticostriatal transmission in an animal model of HD, the current study assessed the impact of systemic administration of PF-04447943 on spontaneous and evoked striatal neuronal activity in aged (9–11 months old) wild-type (WT) and TG5 rats.

## Materials and Methods

### Subjects and Surgery

Subjects were 9–11 month-old wild type (WT) and BACHD transgenic line 5 (TG5) male and female rats. TG5 rats were generated and characterized by Yu-Taeger et al. (2012). Briefly, these transgenic Sprague-Dawley rats express the full-length mutant huntingtin gene with 97 CAG/CAA repeats under the control of the full-length human huntingtin promoter and regulatory elements (Yu-Taeger et al., 2012). TG5 rats at the age range used in the current study exhibit a progressive neuropathology and HD-like phenotype with motor abnormalities that closely resemble early onset HD (Yu-Taeger et al., 2012; Abada et al., 2013; Nagy et al., 2015). Animals were housed under standard conditions (temperature and humidity-controlled, 12-h light/dark cycle) with unrestricted access to food and water. All experiments were performed in accordance with protocols approved by the Rosalind Franklin University Institutional Animal Care and Use Committee. All TG5 rats were genotyped prior to experimentation. Animals were anesthetized with urethane (1.5 g/kg) and placed in a stereotaxic apparatus. Bipolar stimulating electrodes were implanted ipsilaterally into the motor cortex and the substantia nigra pars reticulata (SNr) as described previously (Threlfell et al., 2009; Sammut et al., 2010; Padovan-Neto et al., 2015). A recording electrode was also implanted into the contralateral motor cortex for the monitoring of local field potentials (Tseng et al., 2011). All recordings were performed while the animal was in a slow wave state (delta frequency). Glass extracellular recording electrodes were implanted into the dorsal striatum ipsilateral to cortical and SNr stimulating electrodes. Coordinates for electrode placement from Bregma were as follows: cortex – anterior: 3.7 mm, lateral: 2.0 mm; SNr – posterior: 5.0 mm, lateral: 2.5 mm; striatum – anterior: 0.7–1.2 mm, lateral: 2.0–4.0 mm; ventral from the surface of the brain: cortex: 2.0 mm; SNr: 8.0 mm; striatum: 3.0–7.0 mm (Paxinos and Watson, 2009). At the end of the experiment, rats were perfused with 4% paraformaldehyde and brains were processed for histological examination of electrode placements.

### Electrophysiology

**FIGURE 1:**
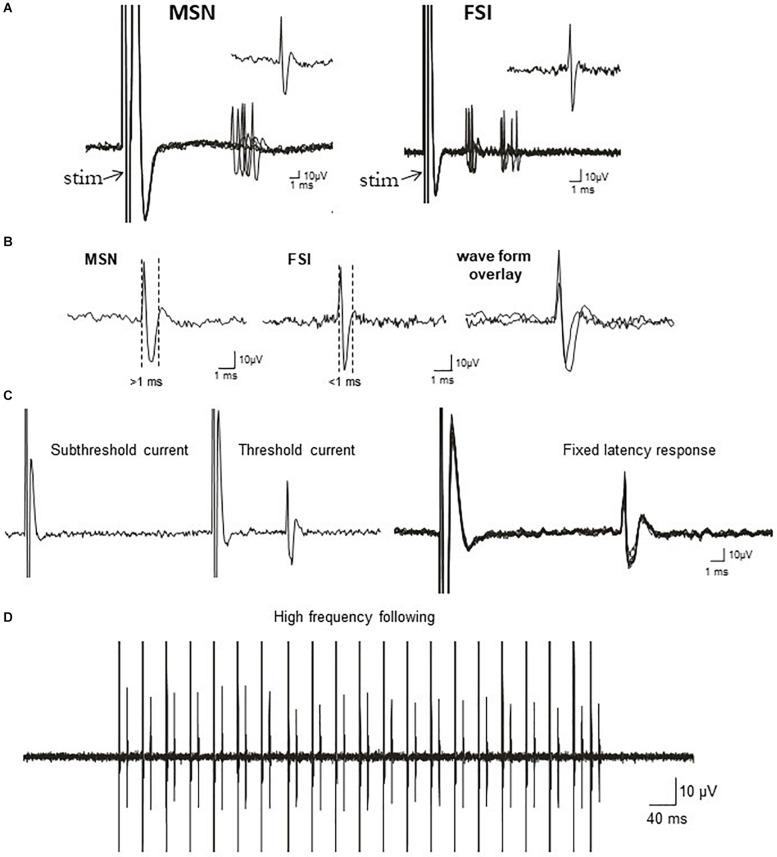
Electrophysiological identification of striatal single-units responding to cortical stimulation: medium-sized spiny projection neurons (MSNs) and fast-spiking interneurons (FSIs). **(A)** Traces show typical cortically-evoked responses from isolated MSNs (*left*) and FSI (*right*). Ten consecutive overlaid responses are shown. Insets show a single event (1 epic) representative of the cortically-evoked responses. FSIs often discharge trains of spikes in response to cortical stimulation, whereas MSNs typically fire in single spikes. **(B)** *Left*: Representative waveform of a cortically-evoked spike from an antidromically-identified striatonigral MSNs (SNr+MSNs). *Middle*: Representative waveform of a cortically-evoked spike from an electrophysiologically-identified FSI. *Right*: Overlay of MSN and FSI waveforms showing the differences in waveform duration between the cell types. **(C)** *Left:* All or none current intensity threshold for activation (responses are never observed at sub-threshold current intensities, but always observed at supra-threshold currents). *Right*: responses always exhibit a fixed latency to SNr stimulation. Ten consecutive overlaid responses are shown. **(D)** Constant fixed latency firing during high frequency antidromic stimulation.

Recording microelectrodes were manufactured from 2.0 mm OD borosilicate glass capillary tubing and filled with 2 M sodium chloride solution (Ondracek et al., 2008). Electrode impendence *in situ* was 15–25 MΩ. The signal to noise ratio for all recordings was ≥4:1. Electrical stimuli (duration = 500 μs, intensity = 400–1,000 μA, in steps of 200 μA and counter-balanced across cells) were generated using a Grass stimulator and delivered in single pulses at 0.5 Hz over 50 consecutive trials via the cortical electrode implanted ipsilateral to the recording pipette. Evoked responses were obtained using AxoScope (version 10.1, Axon Instruments/Molecular Devices) or Neuroscope (version 2.65) software acquired at 20 kHz with a Digidata 1440 A-D converter and Neuro Data IR183A amplifier (Cygnus Technology). Following isolation of single units using cortical stimulation, basal (non-evoked) spike activity and cortical LFPs were recorded for 3 min (Tseng et al., 2011). Cortical stimulation was then delivered in separate trials using a range of stimulus intensities. At the end of each recording, antidromic stimulation of the SNr was attempted to identify a subpopulation of striatonigral (SNr+) projection neurons as previously described (Threlfell et al., 2009; Sammut et al., 2010; Padovan-Neto et al., 2015). The electrophysiological properties of other “putative” MSNs that were not identified as SNr+ were analyzed as a separate group and found to be statistically identical to those positively identified using antidromic activation (Figure 1 and Supplementary Table S1). Putative FSIs were identified based on their characteristic high spontaneous spike discharge rate, unique response to cortical stimulation, and lack of responses to antidromic stimulation of the SNr (Mallet et al., 2005, 2006; Padovan-Neto et al., 2015).

### Compound Administration

The selective PDE9A inhibitor PF-04447943 (3.2 mg/kg) was dissolved in vehicle consisting of 5% (2-Hydroxypropyl)-β-cyclodextrin in physiological saline (Verhoest et al., 2009; Verhoest et al., 2012). This compound and dose have been shown to inhibit PDE9A selectively (IC50 = 8.3 nM, selectivity >1,390 nM over all other PDE isoforms) *in vivo* and elevate striatal cGMP levels by 145%. Drug or vehicle was administered subcutaneously (s.c.) to either WT or TG5 rats at least 20 min prior to recording striatal neuron activity. Recording sessions lasted 30–180 min. Analyses of spike activity following drug administration across time did not reveal time-dependent differences when individual cells were compared. Characterization of neuronal activity in WT rats was performed following vehicle administration, therefore the control groups in Figures 3, 5 are the same.

### Data Analysis

**FIGURE 2:**
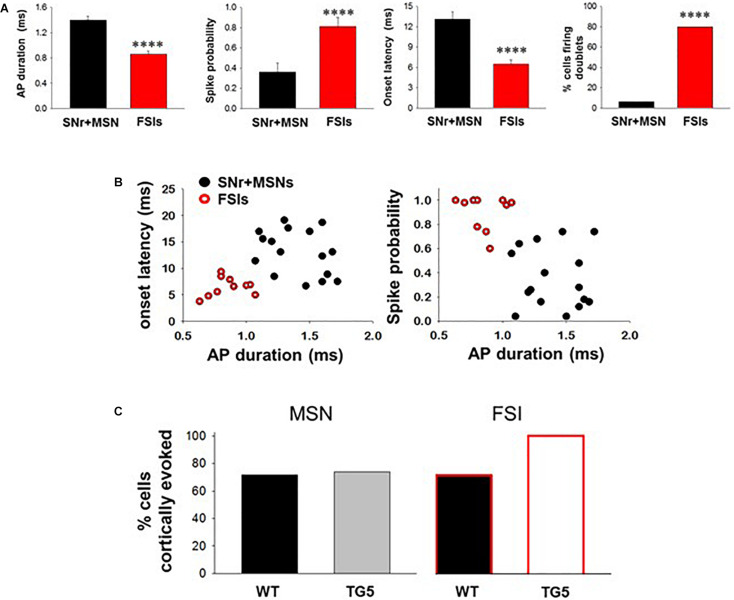
Comparisons between cortically-evoked spike characteristics of striatal MSNs, antidromically-activated MSNs, and FSIs recorded from WT and TG5 HD rats. **(A)** Bar graphs compare the spike duration, probability, onset latency, and number of spike doublets evoked in identified SNr + MSNs and FSIs during cortical stimulation in WT and TG5 rats. Compared with antidromically-activated MSNs, FSIs demonstrate significantly shorter spike duration and onset latency. FSIs also demonstrate significantly higher spike probability and occurrence of doublets compared with SNr + MSNs. Data are presented as mean ± S.E.M. and analyzed using an unpaired *t*-test (^****^p < 0.001). **(B)** Scatter plots of onset latency vs. spike duration (*left*) and spike probability vs. duration (*right*) reveal that SNr+MSNs and FSIs distribute into two separate non-overlapping populations. Data are derived from *n* = 16 SNr+MSNs and *n* = 10 FSIs which responded consistently to cortical stimulation using an intensity of 1,000 μA. **(C)** Graphs show the proportion of striatal neuron subpopulations exhibiting cortically-evoked activity in vehicle-treated WT and TG5 rats. No significant effects of genotype were observed across groups (*p* > 0.05). Data were analyzed using Chi-square tests and are derived from *n* = 46 WT MSNs (*n* = 19 WT rats), *n* = 46 TG5 MSNs (*n* = 19 TG5 rats), *n* = 7 WT FSIs (*n* = 5 WT rats), *n* = 6 TG5 FSIs (*n* = 3 TG5 rats).

Data were analyzed offline using AxoScope (version 10.1, Axon Instruments/Molecular Devices) or Neuroscope 2.65 and Sigma Stat/SigmaPlot 11.0 (Jandel) software. The influence of genotype and drug manipulations on spontaneous and evoked activity of electrophysiologically-identified striatal MSNs and FSIs was determined in between-subjects studies. Firing rate histograms and peri-stimulus time histograms (PSTHs) were constructed (1.0 ms bins) for each recording trial. Action potential (AP) durations were calculated as previously described (Mallet et al., 2005; Padovan-Neto et al., 2015). Spike probability was calculated by dividing the number of evoked APs (0 or 1 per pulse) by the number of stimuli delivered. The onset latency of cortically-evoked spikes was determined from PSTHs as indicated. The statistical significance of genotype- and drug-induced changes in cell activity was determined using either a *t*-test/Mann-Whitney rank sum test, one/two-way ANOVA, or two-way RM ANOVA with a Student Newman-Keuls *post-hoc* analysis. Student *t*-tests were utilized to analyze the differences in spike characteristics between SNr+MSNs and FSIs (Figure 2). Categorical data was analyzed using a Chi-square test as indicated.

## Results

### *In vivo* Characterization of Spontaneously Active and Cortically-Evoked Single-Units in WT and TG5 Rats

The BACHD TG5 rat model of HD exhibits a robust, early onset, and progressive HD-like phenotype that includes the characteristic neuropathological features of the disease accompanied by motor as well as behavioral deficits (Yu-Taeger et al., 2012; Abada et al., 2013). However, it is not known if corticostriatal transmission and activity of striatal FSIs are impaired in intact TG5 rats. Therefore, our initial goal was to characterize any potential differences in corticostriatal transmission and striatal neuron activity in WT and TG5 rats using *in vivo* electrophysiological recording techniques. The number of cells/subjects (n’s) used in studies for each experiment are included in the figure legends. Several studies using *in vivo* recording methods have demonstrated that monosynaptic spike activity in striatal MSNs and FSIs is reliably driven by corticostriatal inputs, and that specific characteristics exhibited by these evoked responses can be utilized to differentiate between these neuronal subtypes (Mallet et al., 2005, 2006; Sharott et al., 2009, 2012; Padovan-Neto et al., 2015). Representative recordings of electrophysiologically identified striatal MSNs and FSIs isolated in WT and TG5 rats during cortical stimulation are shown in Figure 1. Applying similar waveform and cortically-evoked spike analyses as previously described in Mallet et al. (2005, 2006) we identified putative MSNs as having AP durations that are always >1 ms (average ∼ 1.3 ms), whereas FSIs exhibit much shorter duration APs averaging <0.9 ms (Figures 1A,B). Consistent with these reports and in support of our method for striatal projection neuron and FSI identification, the AP duration recorded from evoked single-units in WT and TG5 rats was significantly shorter in identified FSIs compared to antidromically-identified striatonigral (SNr+) MSNs (Figures 1A–D, 2A and Supplementary Tables S1, S2; *p* < 0.001, Mann-Whitney rank sum test). Moreover, SNr+ MSNs and FSIs exhibited differential responses to cortical stimulation, with FSIs responding more robustly (shorter spike onset latency, higher spike probability to 1,000μA stimulation) and often exhibiting responses characterized by spike trains (e.g., doublets, triplets, etc.) (Figures 1A,B, 2A). Further examination of scatter plots of onset latency or spike probability vs. AP duration demonstrate that SNr+MSNs and FSIs distribute into two separate non-overlapping populations (Figure 2B).

No significant effects of genotype on spontaneous firing activity [*F*_(3, 74)_ = 0.757, *p* > 0.05, one-way ANOVA], antidromic responses (*p* > 0.05, *t*-test) or spike characteristics [*F*_(3, 88)_ = 1.333, *p* > 0.05, one-way ANOVA] of unidentified and antidromically-identified (SNr+) MSNs were observed across WT and TG5 rats (Supplementary Table S1). Additionally, no significant effects of genotype on spontaneous firing activity or spike characteristics of electrophysiologically identified FSIs were observed across WT and TG5 rats (Supplementary Table S2; *p* > 0.05, *t*-test). We next examined the relative proportion of MSNs and FSIs exhibiting spontaneous firing and responsiveness to cortical stimulation in WT and TG5 rats (Figure 2C, Supplementary Table S2). The proportion of spontaneously firing and cortically responsive cells was similar across cell types and genotypes (*p* > 0.05, Chi-square test), though we observed an apparent tendency toward an increase in the proportion of cortically responsive FSIs recorded in TG5 rats compared to WT controls (Figure 2C).

### Impact of HD Genotype on Corticostriatal Transmission in MSNs and FSIs

**FIGURE 3:**
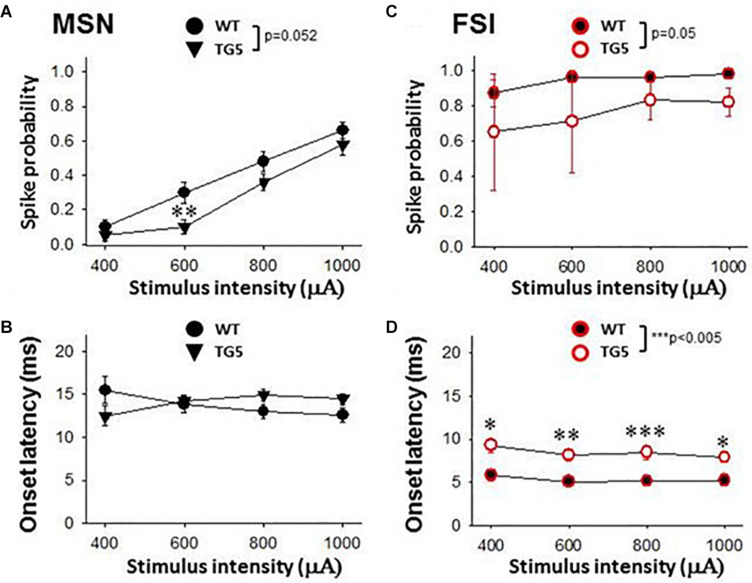
Stimulus-response relationships observed in striatal MSNs and FSIs recorded from WT and TG5 HD rats. Graphs compare spike probability and onset latency of cortically-evoked responses in MSNs and FSIs recorded from WT and TG5 rats. **(A)** Stimulus intensity-dependent effects on cortically-evoked spike probability of MSNs were observed in both WT and TG5 rats (*p* < 0.001). *Post-hoc* comparisons revealed a significant decrease in the probability of evoked responses to cortical stimulation at the 600 μA current intensity in TG5 rats compared with WT rats (**p < 0.01), and a trend toward a decrease at 800 μA (*p* = 0.064). **(B)** No significant differences in onset latency (*p*> 0.05) or SD of latency (data not shown) of cortically-evoked responses were observed in MSNs recorded in TG5 rats as compared to WT controls. **(C)** Cortically-evoked spike probability of FSIs was not significantly affected by increasing current amplitudes (*p* > 0.05). Overall effects of genotype on cortically-evoked spike probability (*p* = 0.05), and **(D)** spike onset latency (***p < 0.005) were observed in FSIs recorded in TG5 rats as compared to WT controls. *Post-hoc* comparisons demonstrated a significant increase in the onset latency of cortically-evoked responses at all current intensities (400–1,000 μA) in FSIs recorded in TG5 rats compared with WT controls (*p < 0.05, **p < 0.01; ***p < 0.005). Data are presented as mean ± S.E.M. and analyzed using two-way RM ANOVA with Student Newman-Keuls *post-hoc* test. Data are derived from *n* = 26 WT MSNs (*n* = 18 WT rats), *n* = 29 TG5 MSNs (*n* = 14 TG5 rats), *n* = 5 WT FSIs (*n* = 3 WT rats), *n* = 6 TG5 FSIs (*n* = 3 TG5 rats).

We next examined stimulus-response relationships of striatal MSNs and FSIs recorded in WT and TG5 rats during low frequency (0.5 Hz) stimulation of the motor cortex (Figure 3). We obtained measures of cortically-evoked spike probability and onset latency in response to a range of different cortical stimulation intensities (400–1,000 μA). Significant effects of stimulus intensity on cortically-evoked spike probability of MSNs were observed in both WT and TG5 rats [Figure 3A; *F*_(3, 157)_ = 111.293, *p* < 0.001, two-way RM ANOVA]. *Post-hoc* comparisons indicated that a strong trend toward an overall effect of genotype on spike probability [Figure 3A; *F*_(1_, _157)_ = 3.937, *p* = 0.052, two-way RM ANOVA]. Moreover, *post-hoc* comparisons revealed a significant decrease in the probability of cortically-evoked responses driven by low to moderate stimulus intensities (600–800 μA) in TG5 rats compared with WT rats (Figure 3A; *p* < 0.01, Student-Newman Keuls *post-hoc* test). There were no significant differences in onset latency (Figure 3B; *p* > 0.05) or SD of latency (data not shown) of cortically-evoked responses in MSNs recorded in TG5 rats as compared to WT controls.

In contrast to the stimulation intensity-dependent effects seen in MSNs, cortically-evoked spike probability of FSIs was not significantly affected by increasing current amplitudes (Figure 3C; *p* > 0.05, two-way RM ANOVA). A significant overall effect of genotype on cortically-evoked spike probability, however, was observed in FSIs recorded in TG5 rats as compared to WT controls [Figure 3C; *F*_(1, 20)_ = 5.099, p = 0.05, two-way RM ANOVA]. Additionally, a more substantial effect of genotype on cortically-evoked spike onset latency was observed in FSIs recorded in TG5 rats as compared to WT controls [Figure 3D; *F*_(1, 19)_ = 16.869, *p* < 0.005, two-way RM ANOVA]. *Post-hoc* comparisons revealed a significant increase in the onset latency of cortically-evoked responses at all current intensities (400–1,000 μA) in FSIs recorded in TG5 rats compared with WT controls (Figure 3D; *p* < 0.05, Student-Newman Keuls *post-hoc* test).

### Impact of PDE9A Inhibition on Spontaneous and Cortically-Evoked MSN Activity in WT and TG5 Rats

**FIGURE 4:**
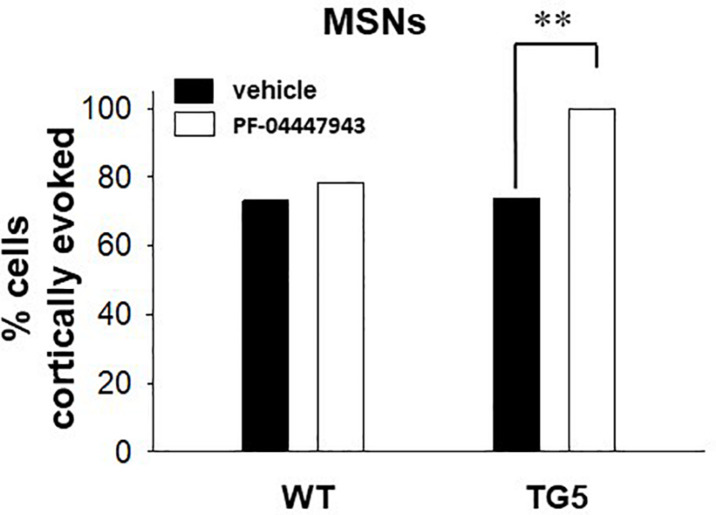
PDE9A inhibition increases the proportion of MSNs responding to cortical stimulation in TG5 HD rats. Bar graphs show the percentage of all recorded striatal MSNs responding to cortical stimulation in WT and TG5 rats following systemic administration of vehicle or the PDE9A inhibitor PF-04447943. A significant increase in the proportion of TG5 MSNs responsive to cortical stimulation was observed after PF-04447943 administration (^∗∗^*p* < 0.01). Data are presented as mean ± SEM and analyzed using a Chi-square test. Data are derived from *n* = 46 WT/vehicle-treated MSNs (*n* = 19 WT rats), *n* = 51 WT/PF-04447943-treated MSNs (*n* = 12 WT rats), *n* = 46 TG5/vehicle-treated MSNs (*n* = 19 TG5 rats), and *n* = 27 TG5/PF-04447943-treated MSNs (*n* = 10 TG5 rats).

To determine whether increases in cGMP levels affects corticostriatal transmission in WT and TG5 rats, we administered vehicle or the selective PDE9A inhibitor PF-04447943, which at the dose used in this study (3.2 mg/kg), produces enduring elevations in cortical and striatal cGMP levels and augment synaptic plasticity in the hippocampus (Verhoest et al., 2009; Hutson et al., 2011; Kleiman et al., 2012). No significant effects of PF-04447943 administration on spontaneous firing activity [*F*_(1, 151)_ = 0.0181, *p* > 0.05, one-way ANOVA] or spike characteristics [*F*_(1, 166)_ = 0.515, *p* > 0.05, two-way ANOVA] of MSNs were observed across WT and TG5 rats (Supplementary Table S2). We next examined the relative proportion of MSNs exhibiting spontaneous firing and responsiveness to cortical stimulation in WT and TG5 rats following vehicle or PF-04447943 administration (Figure 4 and Supplementary Table S2). The proportion of spontaneously firing cells was similar across genotypes and vehicle/drug treatment groups (*p* > 0.05, Chi-square test). We did however, observe a significant increase in the proportion of cortically responsive MSNs recorded in TG5 rats following PF-04447943 treatment compared to vehicle-treated controls [Figure 4; *p* < 0.01, χ^2^_(3)_ = 12.574, *p* < 0.01, Chi-square test], with no significant drug effect in WT rats (*p* > 0.05).

**FIGURE 5:**
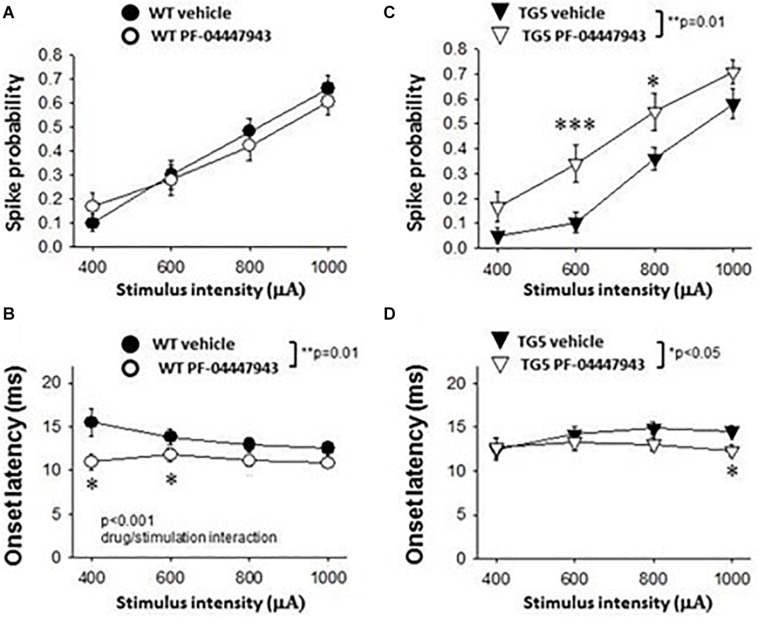
Effects of PDE9A inhibition on stimulus-response relationships of striatal MSNs recorded from WT and TG5 HD rats. Graphs compare cortically-evoked responses in MSNs from the striatum of WT and TG5 rats treated with either vehicle or the selective PDE9A inhibitor PF-04447943 (3.2 mg/kg, s.c.). **(A)** Systemic administration of PF-04447943 did not affect the probability of cortically-evoked spikes in WT rats as compared with vehicle-treated WT controls (*p* > 0.05). **(B)** The onset latency of cortically-evoked spikes was significantly decreased after PF-04447943 administration in WT rats (**p < 0.01). A significant interaction between PF-04447943 treatment and stimulation intensity was also observed (*p* < 0.001). *Post-hoc* comparisons also revealed that PF-04447943 administration decreased the onset latency of cortically-evoked spikes of MSNs at 400 μA and 600 μA (*p < 0.05) stimulus intensities tested in WT rats. A trend toward a decrease in spike latency was also observed at 800 μA (*p* = 0.074) following PF-04447943 administration as compared to vehicle-treated WT rats. **(C)** In contrast to WT rats, an overall effect of PF-04447943 administration on spike probability was observed in MSNs recorded in TG5 rats (**p = 0.01). *Post-hoc* comparisons revealed significant increases in spike probability in TG5 rats at stimulus intensities of 600 μA (***p < 0.005) and 800 μA (*p < 0.05) following PF-04447943 administration as compared to vehicle-treated TG5 rats. **(D)** The onset latency of cortically-evoked spikes was significantly decreased after PF-04447943 administration in TG5 rats (*p < 0.05). *Post-hoc* comparisons revealed significant decreases in onset latency at stimulus intensities of 1,000 μA in TG5 rats treated with PF-04447943 (*p < 0.05). A trend toward a decrease in spike latency was also observed at 800 μA (*p* = 0.072) following PF-04447943 administration as compared to vehicle-treated TG5 rats. There was no change in the standard deviation (SD) of spike latency in WT and TG5 MSNs following PDE9A inhibition (*p* > 0.05, data not shown). Data are presented as mean ± SEM and analyzed using two-way RM ANOVA with Student Newman-Keuls *post-hoc* test. Data are derived from *n* = 26 WT/vehicle-treated MSNs (*n* = 18 WT rats), *n* = 31 WT/PF-04447943-treated MSNs (*n* = 11 WT rats), *n* = 29 TG5/vehicle-treated MSNs (*n* = 14 TG5 rats), and *n* = 21 TG5/PF-04447943-treated MSNs (*n* = 9 TG5 rats).

As shown in Figure 5A, a significant effect of cortical stimulus intensity was observed on the probability of evoking spikes in MSNs in both vehicle- and PF-04447943-treated WT rats [*F*_(3, 165)_ = 94.198, *p* < 0.001, two-way RM ANOVA]. No change in the probability of cortically-evoked spikes was observed between vehicle- and PF-04447943-treated WT MSNs at any stimulus intensity tested (*p* > 0.05, two-way RM ANOVA). However, PF-04447943 administration induced an overall decrease in the onset latency of cortically-evoked spikes in WT rats as compared to vehicle-treated controls [Figure 5B; *F*_(1, 102)_ = 16.536, *p* = 0.01, two-way RM ANOVA]. Moreover, a significant interaction between PF-04447943 treatment and stimulation intensity was observed indicating that PDE9A inhibition facilitates cortically-driven spike generation in MSNs recorded in WT rats in a manner which depends on the recruitment of convergent corticostriatal synaptic inputs [Figure 5B; *F*_(3, 116)_ = 7.903, *p* < 0.001, two-way RM ANOVA]. *Post-hoc* comparisons revealed a significant decrease in the onset latency of cortically-evoked responses at current intensities of 400 μA and 600 μA (*p* < 0.05), and a trend toward a decrease at 800 μA (*p* = 0.074) in MSNs recorded in PF-04447943-treated WT rats compared with vehicle controls (Figure 5B; *p* < 0.05, Student-Newman Keuls *post-hoc* test). The SD of onset latency of cortically-evoked spikes was unaffected by PDE9A inhibition in WT MSNs (*p* > 0.05, data not shown).

**FIGURE 6:**
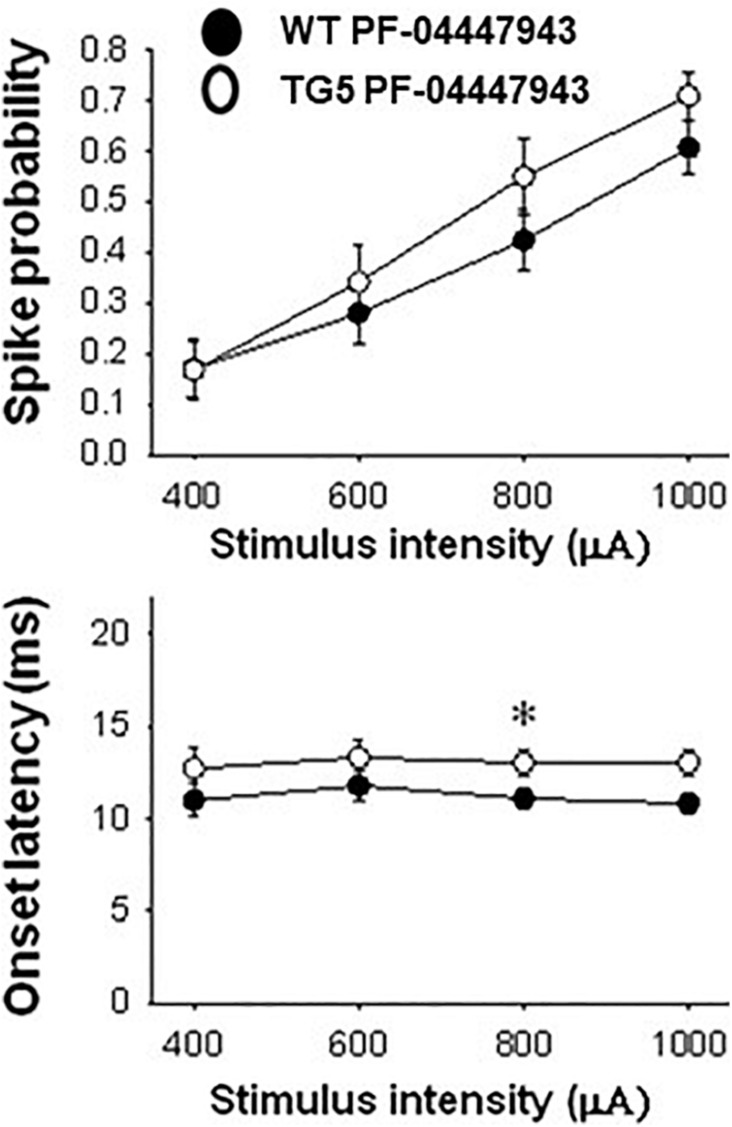
Comparison of stimulus-response relationships observed in MSNs recorded from WT and TG5 rats treated with the selective PDE9A inhibitor. Top: The increase in the mean ± S.E.M. probability of cortically-evoked spikes recorded from MSNs in WT rats following PF-04447943 (3.2 mg/kg, s.c.) administration was similar to measures made in TG5 rats [*p* > 0.05, *F*_(1, 200)_ = 2.563; *n* = 31 WT/PF-04447943 and *n* = 21 TG5/PF-04447943-treated MSNs]. A significant effect of stimulation intensity was observed in both groups [*p* < 0.001, *F*_(3, 200)_ = 22.674]. Bottom: The mean ± S.E.M. onset latency of cortically-evoked spikes recorded from MSNs in WT rats was significantly decreased following PF-04447943 (3.2 mg/kg, s.c.) administration [*p* < 0.005, *F*_(1, 157)_ = 9.040, two-way ANOVA with Student-Newman-Keuls *post-hoc* test] as compared to measures taken in PF-04447943-treated TG5 rats (*n* = 31 WT/ and *n* = 21 TG5 PF-04447943-treated MSNs). *Post-hoc* comparisons revealed significant decreases in onset latency of cortically-evoked spikes for trials using 0.8 mA (*p < 0.05) stimulus intensities in WT rats.

In TG5 rats, a significant effect of stimulus intensity on cortically-evoked spike probability was observed in both vehicle- and PF-04447943-treated groups [*F*_(3, 142)_ = 85.226, *p* < 0.001, two-way RM ANOVA]. Interestingly, PF-04447943 administration produced robust facilitatory effects on cortically-evoked spike probability in TG5 rats compared with vehicle-treated controls [Figure 5C; *F*_(1, 142)_ = 7.147, p = 0.01, two-way RM ANOVA]. *Post-hoc* comparisons revealed significant increases in spike probability at 600 μA (*p* < 0.005) and 800 μA (*p* < 0.05). The onset latency of cortically-evoked responses was also decreased in PF-04447943-treated TG5 rats compared to vehicle-treated controls [Figure 5D, *F*_(1, 82)_ = 6.197, *p* < 0.05, two-way RM ANOVA]. *Post-hoc* comparisons revealed significant PF-04447943-induced decreases in cortically-evoked spike onset latency at 1,000 μA (*p* < 0.05) in MSNs recorded in TG5 rats compared with vehicle-treated controls (Figure 5D; *p* < 0.05, Student-Newman Keuls *post-hoc* test). A trend toward a PF-04447943-induced decrease in cortically-evoked spike onset latency was also observed at 800 μA stimulus intensities (*p* = 0.072). The SD of onset latency of cortically-evoked spikes was unchanged by PDE9A inhibition in TG5 MSNs (*p* > 0.05, data not shown). Lastly, comparisons of stimulus-response relationships observed in MSNs recorded from WT and TG5 rats treated with the selective PDE9A inhibitor showed similar effects on probability of cortically-evoked spikes (Figure 6 Top; *p* > 0.05). However, drug treatment had a modest but significant facilitatory effect on onset latency in WT striatal MSNs as compared to TG5 MSNs during trials using 0.8 mA (Figure 6 Bottom; ^∗^*p* < 0.05) stimulus intensities in WT rats.

## Discussion

The current observations demonstrate that TG5 HD rats (9–11 months of age) exhibit significant abnormalities in corticostriatal transmission as determined by measures of cortically-evoked spike activity in MSNs and FSIs. Interestingly, FSI activation by corticostriatal inputs was more robustly affected in TG5 rats as indicated by large increases in the onset latency and significant overall decreases in the probability of cortically-evoked spikes. Inhibition of PDE9A using the potent and selective compound PF-04447943 decreased the onset latency of cortically-evoked spikes in MSNs in both WT and TG5 rats, indicating that this compound facilitates corticostriatal transmission likely via augmentation of striatal cGMP signaling (Padovan-Neto et al., 2015). Moreover, PDE9A inhibition robustly increased the proportion of MSNs responding to cortical stimulation and reversed deficits in spike probability in TG5 rats. Thus, PDE9A inhibition and elevations in intracellular cGMP may augment the responsiveness of TG5 MSNs to cortical drive and recruit a quiescent or less excitable subpopulation of cells which may be compromised by the expression of mutant huntingtin. The effectiveness of PDE9A inhibition for increasing cortically-driven activity in the TG5 rat striatum supports the targeting of PDE9A or other cGMP effector proteins (e.g., PKG, CNGCs) for therapeutic intervention in HD.

### Pathological Changes in Corticostriatal Drive in TG5 Rats

The later stages of HD (grade 3–4) are characterized by increasing dysfunction of the motor and premotor cortices, as well as progressive striatal MSN and GABAergic interneuron cell death or dysregulation (Reiner et al., 1988, 2013; Aylward et al., 1998; Macdonald and Halliday, 2002; Rosas et al., 2003; Vonsattel et al., 2008; Holley et al., 2019). Studies in genetic rodent models of HD have demonstrated that expression of mutant huntingtin and progressive dysfunction of frontal-subcortical networks induces complex changes at the cellular and systems levels including decreases in glutamatergic corticostriatal transmission and synchronous MSN burst firing, aberrant synaptic plasticity, and increases in intrinsic membrane excitability of MSNs and a subpopulation of GABAergic interneurons (Cepeda et al., 2007; Bunner and Rebec, 2016; Puigdellivol et al., 2016; Raymond, 2017; Holley et al., 2019). Additionally, morphological abnormalities including reductions in cortical and striatal volume and decreases in spine density of MSNs are present at relatively early stages in mouse models of HD, which in some cases (e.g., the R6/2 mouse), is followed by late onset neurodegeneration (Turmaine et al., 2000; Klapstein et al., 2001; Levine et al., 2004; Raymond et al., 2011; Heikkinen et al., 2012; Indersmitten et al., 2015; Puigdellivol et al., 2016; Patel et al., 2018). In most HD models, this pathology is associated with motor abnormalities such as early hyperactivity followed by progressive hypoactivity in the open field, as well as coordination, balance, and gait deficits (Hickey et al., 2008; Pouladi et al., 2013).

In the TG5 rat model, progressive rotarod deficits, hindlimb clasping, gait abnormalities and other motor and non-motor deficits emerge between 1 and 12 months of age (Yu-Taeger et al., 2012; Abada et al., 2013; Nagy et al., 2015). Additionally, by 1 year of age these animals exhibit considerable neuropathology in the cortex, striatum and other brain regions including neuropil aggregates, mutant huntingtin deposits in axons, synaptic terminals and cell nuclei, and decreases in brain volume and striosome area (Yu-Taeger et al., 2012). Compared to WT controls, TG5 rats also exhibit lower levels of hippocampal and cortical field potential power in the gamma frequency, indicative of compromised GABAergic interneuron activity in these networks (Nagy et al., 2015).

Consistent with the above reports, the most striking effects of the TG5 genotype observed in the current study were deficits in corticostriatal transmission particularly in recorded FSIs (i.e., decreased spike probability and increased spike onset latency compared to WT FSIs). MSNs were also less responsive to cortical stimulation in TG5 rats, although this effect was more modest. These observations suggest that FSIs may be preferentially affected and more dysfunctional than MSNs in the TG5 model at the age range examined in this study. This finding is consistent with studies by Reiner et al. (2013) showing that large and progressive decreases in striatal parvalbumin-expressing FSIs are observed in post-mortem studies of HD patients. Thus, loss or dysfunction of glutamatergic input to FSIs in TG5 rats may precede degeneration of these interneurons, potentially resulting from BDNF deficiency or calcium dysregulation (Agerman and Ernfors, 2003; Raymond, 2017). Given that FSIs recorded *in vivo* are considerably more active and responsive to cortical drive than MSNs (Mallet et al., 2005) they could potentially, be more sensitive to pathological insults resulting from the HD mutation.

In contrast to our findings of decreased cortical drive on FSIs in TG5 rats, previous studies have reported increases in spontaneous IPSCs and the IPSC/EPSC ratios recorded in MSNs in several models of HD (Cummings et al., 2009; Raymond et al., 2011; Dvorzhak et al., 2013; Indersmitten et al., 2015). These observations suggest that GABAergic drive and feedforward inhibition is elevated in HD, possibly due to increased spontaneous FSI activity. Currently, however, the source of this GABAergic inhibition is unclear as a variety of other striatal interneurons (NPY, calretinin, and dopamine synthesizing intrinsic cells) contribute to feedforward inhibition, and MSNs also participate in collateral GABAergic inhibition of adjacent MSNs (Tepper et al., 2004; Zhai et al., 2018). The later source of GABAergic inhibition is implicated based on observations that the spontaneous firing of MSNs is abnormally elevated in many HD models (Miller et al., 2010; Bunner and Rebec, 2016; Raymond, 2017). We did find some evidence for elevated basal firing of both MSNs and FSIs in TG5 rats in the current study, however, the small numbers of slow spontaneously firing neurons in the anesthetized preparation likely limited the power of these group comparisons (Rebec et al., 2006). The contribution of cortically-driven vs. spontaneous firing activity of FSIs and other GABAergic neurons to the increase in GABAergic inhibition of MSNs in the HD striatum needs to be assessed further in brain slice preparations.

### Impact of PDE9A Inhibition on Corticostriatal Transmission in MSNs Recorded in WT and TG5 Rats

Disruptions in striatal cyclic nucleotide signaling and metabolism have been frequently reported in rodent models of HD (Cramer et al., 1981, 1984; Luthi-Carter et al., 2000; Gines et al., 2003; Hebb et al., 2004; Hu et al., 2004; Leuti et al., 2013; Beaumont et al., 2016). Moreover, decreases in hippocampal and striatal neuronal nNOS mRNA and protein, and cGMP levels are observed in the R6/1 and R6/2 HD models (Deckel et al., 2001, 2002; Perez-Severiano et al., 2002; Zucker et al., 2005; Cipriani et al., 2008; Padovan-Neto et al., 2019) as well as in post-mortem tissue from patients with HD (Morton et al., 1993; Norris et al., 1996; Saavedra et al., 2013). Recent studies also indicate that PDE9A may be compartmentalized proximal to the particulate isoform of GC (pGC) which is associated with the cellular membrane and activated by peptides and not NO (Harms et al., 2019). This intracellular pool of cGMP may be anatomically and functionally distinct from the canonical NO driven sGC-cGMP pathway and as such, may represent an additional target for treatment of disorders such as HD. Irregardless of the source of cGMP tone, pharmacotherapies such as PDE inhibitors designed to target and normalize the function of these signaling pathways are of high clinical significance for treating motor and cognitive symptoms in patients with HD (Wild and Tabrizi, 2014; Mrzljak and Munoz-Sanjuan, 2015; Puigdellivol et al., 2016). In support of this, the PDE5 inhibitor sildenafil was shown to increase hippocampal cGMP levels and improve novel object recognition memory and passive avoidance learning in R6/1 mice (Puigdellivol et al., 2016). PDE10A inhibition was also found to facilitate corticostriatal transmission, rescue hippocampal LTP, and reverse basal ganglia dysfunction in the R6/2 and Q175 mouse models of HD via cAMP- and cGMP-dependent mechanisms (Beaumont et al., 2016). Chronic PDE10 inhibitor administration has also been reported to attenuate cortical and striatal neurodegeneration, improve motor behavior, increase life-span, and elevate activated CREB in MSNs in the quinolinic acid and R6/2 models of HD (Giampa et al., 2009a, Giampa et al., 2010).

Unfortunately, recent neuroimaging studies have reported decreases in the levels of several PDE isoforms in the striatum of rodent models of HD (Beaumont et al., 2016) as well as in patients with HD (Ahmad et al., 2014; Russell et al., 2014), complicating the consideration of their utility as targets for novel therapeutics for HD. Decreased levels of PDEs such as PDE1B and PDE10A are shown to coincide with locomotor as well as learning and memory deficits (Reed et al., 2002; Siuciak et al., 2006). Various striatal PDE isoforms are likely to be compartmentalized and differentially targeted to pre-, post-, and/or peri-synaptic sites (Xie et al., 2006; Nishi et al., 2008), which may explain why specific PDEs such as PDE10A are preferentially compromised in HD models (Hebb et al., 2004; Beaumont et al., 2016; Patel et al., 2018). Other PDE isoforms including PDE5 and PDE9A are preserved in HD, and as a result, may be better therapeutic targets (Saavedra et al., 2013; Puigdellivol et al., 2016). A further consideration is the locus or loci of the PDE9A inhibitor effects, which are likely not exclusive to the striatum but may involve frontal cortical regions and limbic regions which also express PDE9A such as the lateral septum and hippocampus (Andreeva et al., 2001; Nagy et al., 2015). These regions innervate striatum and have been implicated in the behavioral effects of PDE9A inhibitors (González-Maeso et al., 2007; Hutson et al., 2011; Kleiman et al., 2012).

Interestingly, the pattern of PDE9A expression in frontal-subcortical circuits closely resembles that of nNOS and sGC (Andreeva et al., 2001; Vincent, 2010) suggesting a possible functional association between cGMP-mediated regulation of excitatory synaptic transmission and generation of purposeful movement, motivational processes, cognition, and learning (Hutson et al., 2011; Kleiman et al., 2012; Threlfell and West, 2013). In the current study, administration of the selective PDE9A inhibitor PF-04447943, using a well-characterized dose shown to robustly increase striatal cGMP levels (Verhoest et al., 2009; Kleiman et al., 2012), was found to produce a potent increase in responsiveness of MSNs to cortical drive in both WT and TG5 rats. In WT rats, more significant changes were observed in measures of onset latency, whereas in the TG5 rats more robust facilitatory effects on spike probability were observed. These data indicate that PDE9A inhibition can be effective for increasing responsiveness of MSNs to cortical drive in both the intact and diseased HD striatum, however, the underlying mechanism leading to the observed facilitation may be different (i.e., presynaptic vs. postsynaptic effects, or direct effects on corticostriatal glutamatergic drive at the level of the cortex). Future studies will need to determine the site of cGMP modulation responsible for the observed outcomes and assess further how increased cGMP tone induced following PDE9A inhibition affects striatal and cortical processing in other HD models.

## Conclusion

The current study shows for the first time that acute systemic exposure to the PDE9A inhibitor PF-04447943 results in robust increases in MSN responsiveness to cortical drive in TG5 rats. The effect of PF-04447943 completely reversed deficits in corticostriatal transmission in MSNs induced by mutant huntingtin and increased the population of MSNs responding to cortical stimulation, suggestion that this pharmacotherapy may be efficacious in more advanced stages of HD and in more robust HD models. These results also support previous observations indicating that corticostriatal circuits are compromised in HD in a manner involving abnormalities in cyclic nucleotide-PDE signaling cascades (Beaumont et al., 2016). Pathological changes in intracellular cGMP signaling cascades may alter the way MSNs respond to corticostriatal input and/or neuromodulation by dopamine and NO (Threlfell and West, 2013). It is also possible that augmentation of cGMP/PKG signaling by PF-04447943 could lead to facilitatory effects on downstream targets such DARPP-32 which may offset deficits in cAMP/PKA signaling (Nishi et al., 2008). Thus, augmentation of cGMP could be useful for restoring aberrant cyclic nucleotide tone and DARPP-32 signaling in the HD striatum.

## Data Availability Statement

All datasets generated for this study are included in the article/Supplementary Material.

## Ethics Statement

The animal study was reviewed and approved by Rosalind Franklin University Institutional Animal Care and Use Committee.

## Author Contributions

AW, SC, AD, and PC performed and analyzed all *in vivo* electrophysiology experiments described herein. VB coordinated research activities by providing drug dosing information and access to the Pfizer compound and BACHD rats. AW and VB designed all experiments and acquired the financial support for these studies. AW, SC, GS, and FM provided intellectual contributions and collectively wrote the manuscript.

## Conflict of Interest

VB was employed by CHDI Foundation Inc. The remaining authors declare that the research was conducted in the absence of any commercial or financial relationships that could be construed as a potential conflict of interest.
