# Supplementary material for: Phosphodiesterase 9A Inhibition Facilitates Corticostriatal Transmission in Wild-Type and Transgenic Rats That Model Huntington’s Disease
Source: Front Neurosci. 2020 Jun 3;14:466. doi: 10.3389/fnins.2020.00466 (PMC7283904; doi:10.3389/fnins.2020.00466)
Supplement: Supplementary file 1 [file Table_1.pdf]

Supplemental Table 1. Comparison of spike properties of unidentified MSNs and identified SNr+ MSNs across genotypes.

	**Group**	**Firing rate (Hz)**	**Spike duration (ms)**	**Antidromic spike threshold (µA)**	**Antidromic spike latency (ms)**
**Unidentified MSNs**	WT-veh (29 cells)	0.0051 ± 0.003	1.28 ± 0.026	na	na
	TG5-veh (32 cells)	0.0814 ± 0.052	1.37 ± 0.033	na	na
**Identified SNr+ MSNs**	WT-veh (17 cells)	0.0000 ± 0.000	1.33 ± 0.046	858 ± 111	9.9 + 0.9
	TG5-veh (14 cells)	0.0020 ± 0.002	1.32 ± 0.061	634 ± 92	9.4 + 0.9

Data are presented as mean ± SEM. Data were analyzed using one-way ANOVA (all MSNs) and t-test (SNr+ MSNs). $p>0.05$ for all comparisons. Abbreviations: MSN, medium-sized spiny neuron; TG5, BAC transgenic HD rats; veh, vehicle; WT, wild-type rats.
