# Supplementary material for: Phosphodiesterase 9A Inhibition Facilitates Corticostriatal Transmission in Wild-Type and Transgenic Rats That Model Huntington’s Disease
Source: Front Neurosci. 2020 Jun 3;14:466. doi: 10.3389/fnins.2020.00466 (PMC7283904; doi:10.3389/fnins.2020.00466)
Supplement: Supplementary file 2 [file Table_2.pdf]

Supplemental Table 2. Comparison of spontaneous firing activity of striatal MSNs and FSIs across genotypes and drug treatments.

	**Group**	**Firing rate (Hz)**	**Spike duration (ms)**	**% cells firing**
**Spontaneously active and quiescent MSNs**	WT-veh (46 cells)	0.0031± 0.002	1.30 ± 0.024	7.3%
	TG5-veh (46 cells)	0.058 ± 0.052	1.35 ± 0.030	10.8%
	WT-PDE9Ai (51cells)	0.012 ± 0.006	1.37 ± 0.025	13.7%
	TG5-PDE9Ai (27 cells)	0.057 ± 0.045	1.32 ± 0.026	14.8%
**Spontaneously active and quiescent FSIs**	WT-veh (7 cells)	4.25 ± 2.88	0.89 ± 0.064	71.4%
	TG5-veh (6 cells)	7.74 ± 7.74	0.86 ± 0.033	20.0%

Data are presented as mean ± SEM. Data were analyzed using Two-way ANOVA (MSNs) and t-test (FSIs). $p>0.05$ for all comparisons. Abbreviations: FSI, fast-spiking interneuron; MSN, medium-sized spiny neuron; PDE9Ai, phosphodiesterase 9A inhibitor (PF-04447943), TG5, BAC transgenic HD rats; veh, vehicle; WT, wild-type rats.
